# Shear Bond Strength of Al_2_O_3_ Sandblasted Y-TZP Ceramic to the Orthodontic Metal Bracket

**DOI:** 10.3390/ma10020148

**Published:** 2017-02-09

**Authors:** Seon Mi Byeon, Min Ho Lee, Tae Sung Bae

**Affiliations:** 1 Department of Dental Biomaterials and Institute of Biodegradable Material, Institute of Oral Bioscience and BK21 Plus Project, School of Dentistry, Chonbuk National University, 567 Baeckje-daero, Deokjin-gu, Jeonju-si, Jeollabuk-do 54896, Korea; seonmi793@gmail.com (S.M.B.); mh@jbnu.ac.kr (M.H.L.); 2 Research Institute of Clinical Medicine of Chonbuk National University, Biomedical Research Institute of Chonbuk National University Hospital, 20 Geonji-ro, Deokjin-gu, Jeonju-si, Jeollabuk-do 54907, Korea

**Keywords:** yttria-stabilized tetragonal zirconia polycrystal (Y-TZP), shear bond strength, alumina sandblasting, silane primer, MDP-based primer, resin cement

## Abstract

As the proportion of adult orthodontic treatment increases, mainly for aesthetic reasons, orthodontic brackets are directly attached to yttria-stabilized tetragonal zirconia polycrystal (Y-TZP) restorations. This, study analyzed the shear bond strength (SBS) between various surface treated Y-TZP and orthodontic metal brackets. The Y-TZP specimens were conditioned by 110 μm Al_2_O_3_ sandblasting, or sandblasting followed by coating with one of the primers (silane, MDP, or an MDP-containing silane primer). After surface treatment, the orthodontic metal bracket was bonded to the specimen using a resin cement, and then 24 h storage in water and thermal cycling (5000 cycles, 5–55 °C), SBS was measured. Surface roughness was analyzed for surface morphology, and X-ray photoelectron spectroscopy (XPS) was employed for characterization of the chemical bond between the Y-TZP and the MDP-based primers (MDP, MDP containing silane primer). It was found that after surface treatment, the surface roughness of all groups increased. The groups treated with 110 μm Al_2_O_3_ sandblasting and MDP, or MDP-containing silane primer showed the highest SBS values, at 11.92 ± 1.51 MPa and 13.36 ± 2.31 MPa, respectively. The SBS values significantly decreased in all the groups after thermal cycling. Results from XPS analysis demonstrated the presence of chemical bonds between Y-TZP and MDP. Thus, the application of MDP-based primers after Al_2_O_3_ sandblasting enhances the resin bond strength between Y-TZP and the orthodontic metal bracket. However, bonding durability of all the surface-treated groups decreased after thermal cycling.

## 1. Introduction

Recently, several studies have reported the importance of the bond strength between yttria-stabilized tetragonal zirconia polycrystal (Y-TZP) ceramics and resin cement [1,2,3,4,5]. Because of its aesthetic, excellent biocompatibility and superior mechanical properties, Y-TZP-based restoratives are widely used in dental clinics [6,7,8,9]. With the introduction of CAD (computer-aided design)/CAM (computer-aided manufacturing) methods for the production of dental restoratives, the designing of teeth shapes has become more refined. Additionally, it has been possible to reduce the production time of restoratives and the frequency of patient visits to dental clinics [10,11]. The development of staining techniques has enabled the reproduction of tooth-matched colors. Thus, Y-TZP based full-contour all zirconia crowns and bridges have been widely utilized [12,13].

With increasing use of Y-TZP-based restoratives, surface treatment methods are required to enhance the bond strength between the orthodontic bracket and the Y-TZP ceramic for patients who need to undergo orthodontic treatment. Because of the high stability, the Y-TZP ceramic does not easily bond to the resin. Therefore, a method to enhance this bond strength is required, unlike the case where bonding occurs between the bracket and teeth [14]. To enhance the bond strength of the bracket to Y-TZP, it is important to maintain the bonding between the Y-TZP ceramic and the resin [15,16]. As surface treatment methods become more complicated, the clinical application of these methods become more difficult, which means that finding a simple method to enhance the bond strength becomes imperative. To increase the bond strength, a variety of surface treatments [17,18,19] such as rough polishing, sandblasting with high strength particles, glass coating, acid etching, laser treatment, and primer treatments have been reported [14,20,21,22,23].

Micromechanical retention is largely associated with the surface microstructure of the restoratives, which bond with the resin cement. Surface roughness affects the bond strength, and a rougher surface with a larger surface area increases micro-retention for the resin cement [24]. The sandblasting method, which involves blasting with alumina particles, produces micro-unevenness. However, this method has the limitation that it increases the surface area and thus leads to limited improvement in bond strength [25,26]. Representative chemical bonding methods such as the primer treatments, including silane (3-methacryloxypropyl trimethoxy-silane, MPS) or 10-methacryloyloxydecyl dihydrogen phosphate (MDP), have been used [27,28]. Silane primers improve the chemical bond with resin in silica-based feldspathic porcelain, leucite, or lithium disilicate crystal-based glass ceramics. However, it is difficult for Y-TZP to increase the bond strength due to its high chemical stability and because it does not contain silica [29,30]. Phosphate ester included in MDP-based primers can form a chemical bond to the hydroxyl groups on the surface of zirconia [31]. Iwasaki et al. [32] measured the bond strength between an indirect composite and the zirconia framework using a variety of primers, and Lee et al. [33] used different types of primers to enhance the bond strength between the orthodontic bracket and glazed zirconia.

In addition, thermal cycling (TC) was conducted to understand the effect of water exposure and temperature change in the oral environment on the bond strength between Y-TZP and the bracket [34,35] for different numbers of cycles ranging from 500 to 100,000 at temperatures between 5 and 55 °C [14]. For long-term water storage, the effect of the various treatment conditions on the bond strength was studied at the average temperature in the oral environment for a time period of 3–12 months [34,36,37].

Generally, methods such as shear bond strength and tensile bond strength tests have been used to measure the bond strength [38]. The shear bond strength (SBS) test is commonly used to measure the bond strength and in parallel, an analysis of the failure modes in the bonding area is also conducted [39].

In this study, we used silane, MDP, and MDP containing silane primers after alumina sandblasting to enhance the resin bond strength between the Y-TZP ceramic and the orthodontic metal bracket. In addition, we measured the SBS and analyzed the failure modes to measure the strength of bonding to the resin, and analyzed the effect of surface roughness. We also conducted X-ray photoelectron spectroscopy (XPS) to identify the chemical bond between Y-TZP and all the primers.

## 2. Materials and Methods

### 2.1. Preparation of Specimens and Surface Treatments

One hundred and thirty cuboid shaped ceramic specimens were cut from a pre-sintered yttria-stabilized tetragonal zirconia polycrystal (Y-TZP) ceramic block (Zirtooth, HASS, Gangneung, Korea) in distilled water using a high speed diamond saw (Isomet 5000, Buehler, Lake Bluff, IL, USA). The cuboid shaped Y-TZP was sintered according to the manufacturer’s instructions (the final size for shear bond strength test was 8 × 8 × 4 mm^3^ (*n* = 100) and that for surface characterization was 8 × 8 × 2 mm^3^ (*n* = 30)).

The bonding surface of Y-TZP was polished with a # 400–1200 SiC sandpaper, washed by sonicating for 10 min in distilled water, and dried. Next, the bonding surface of Y-TZP was sandblasted with 110 μm alumina (Al_2_O_3_) particles from a distance of 10 mm for 10 s under 0.3 MPa at a constant impact angle of 90°. The sandblasted Y-TZP was heated at 1200 °C for 10 min to stabilize the monoclinic phase to the tetragonal phase. Next, the silane primer (ESPE Sil, 3M ESPE, St. Paul, MN, USA), the MDP primer (Z-PRIME Plus, Bisco, Schaumburg, IL, USA), or the MDP containing silane primer (Clearfil Ceramic primer, Kuraray Medical, Tokyo, Japan) was applied on the sandblasted Y-TZP with a disposable brush tip. The entire primer treated surface was sufficiently dried by blowing mild oil-free air.

The MDP primer was applied to increase the bond strength between the metal bracket for maxillary central incisor (Archist Bracket, Daeseung medical, Seoul, Korea) and the resin cement, after which, the bracket was bonded to the surface treated Y-TZP under a force of about 5 N [40] using the resin cement (Transbond XT, 3M Unitek, Monorvia, CA, USA). Excess resin cement was removed with an explorer, and the remaining resin cement was light cured for 20 s on each side using a halogen light curing unit (Demetron Optilux, Kerr model-VCL 401, Demetron Research, Danbury, CT, USA) at an angle of 45°. The irradiance of light curing was measured with a curing radiometer (Model 100, Demetron Research, Danbury, CT, USA) and found to be >400 mW/cm^2^.

Fifty of the metal bracket bonded Y-TZP were stored in distilled water at 37 ± 1 °C for 24 h. The other fifty were thermal-cycled (INV-TCS-109, Invertech, Gwangju, Korea) in distilled water at 5 °C and 55 °C for 5000 times with a dwell and transfer times of 15 s, each.

Based on the surface treatments and thermal cycling protocol, the bracket bonded Y-TZP was divided randomly into eight groups, as given in Table 1. The materials used in the present study are listed in Table 2.

### 2.2. Measurement of Surface Roughness

To compare the surface roughness of the Y-TZP after the surface treatments, it was analyzed using a surface roughness tester (SV-3000, Mitutoyo, Tokyo, Japan) (3 per non-thermal cycled group). The surface roughness tester was used with the diamond stylus to move along a length of 5 mm at a speed of 0.2 mm/s.

### 2.3. Surface Morphology and Characterization

The morphology of Y-TZP after the different surface treatments (one sample per group without thermal cycling) was observed by both field emission scanning electron microscopy (FE-SEM; SU-70, Hitachi, Tokyo, Japan) and atomic force microscopy (AFM; Multimade-8, Bruker, Billerica, MA, USA).

The surface treated Y-TZP was analyzed using energy dispersive X-ray spectroscopy (EDS; EDAX Octane pro, Ametek, NJ, USA) to characterize the distribution of elemental composition and EDS detection.

### 2.4. Chemical Bond Characterization

In order to demonstrate the chemical bond between the Y-TZP ceramic and MDP containing primer, Y-TZP treated with MDP containing primers were detected using X-ray photoelectron spectroscopy (XPS; Theta Probe AR-XPS System, Thermo Fisher Scientific, Runcorn, UK) (1 per non-thermal cycled group). The samples were placed in a μ-metal ultra-high vacuum (UHV) chamber of an X-ray photoelectron spectrometer. The data were analyzed under the following conditions: monochromatic Al-Kα radiation source (1486.6 eV), X-ray beam diameter (400 μm), and X-ray energy (15 kv, 150 W).

### 2.5. Shear Bond Strength (SBS) Test 

All of the metal bracket bonded Y-TZP was subjected to SBS test using a universal testing machine (Model 4201, Instron, Canton, MA, USA) (10 per group). The bracket bonded Y-TZP was fixed to the jig and subjected to shear force with a chisel shaped metal rod, at a crosshead speed of 1.0 mm/min.

Measured the load at the metal bracket failed from the Y-TZP was calculated by following equation for the SBS value: Load at fracture (N)/Surface area of bracket provided by the manufacturer (8.05 mm^2^).

### 2.6. Evaluation of Failure Modes

After measuring the SBS, the bonded surface were analyzed using a light microscope (DM 2500M, Leica Microsystems, Wetzlar, Germany) at a magnification of 20× to examine the failure modes between the Y-TZP and the orthodontic metal bracket. The failure modes were then classified into adhesive failure, cohesive failure, or mixed (adhesive + cohesive) failure, and the ratios of the failure modes were calculated. Next, the failed surfaces of the Y-TZP and the metal brackets were analyzed using SEM (JSM-6400, JEOL, Tokyo, Japan).

### 2.7. Statistical Analysis

Statistical processing was performed using SPSS 12.0 software (SPSS, Chicago, IL, USA). After the experiment, a one-way ANOVA (ANalysis Of VAriance) test was performed, and variables showing significant differences were analyzed by Tukey’s combined comparison test (*p* value = 0.05).

## 3. Results

### 3.1. Shear Bond Strength (SBS) and Failure Modes

**Figure 1:**
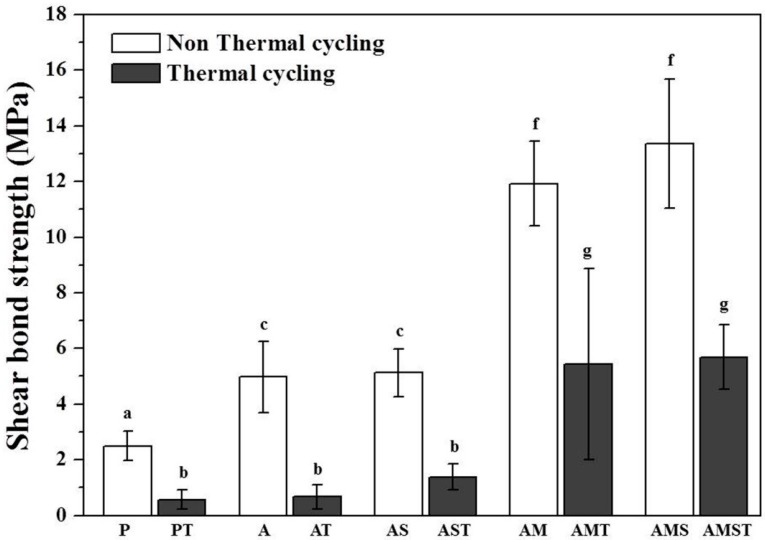
Shear bond strength (SBS) of an orthodontic metal bracket bonded to Y-TZP after surface treatments with and without thermal cycling. Bars indicate the standard deviation. a–g: Groups denoted by different letters are significantly different (*p* < 0.05).

Figure 1 shows the SBS values (means and standard deviation) between the surface treated Y-TZP and the bracket before and after thermal cycling (aging). Compared to the unaged groups (P, A, AS, AM, and AMS), the artificially aged groups (PT, AT, AST, AMT, and AMST) had significantly lower SBS values (*p* < 0.05). The AM (11.92 ± 1.51 MPa) and AMS (13.36 ± 2.31 MPa) groups showed higher SBS values than the other groups (*p* < 0.05). There was no significant difference between A (4.98 ± 1.28 MPa) and AS (5.13 ± 0.85 MPa) groups (*p* > 0.05). Comparing the groups after thermal cycling, it was found that there was no significant difference between PT, AT, and AST, or between AMT and AMST groups (*p* > 0.05).

**Figure 2:**
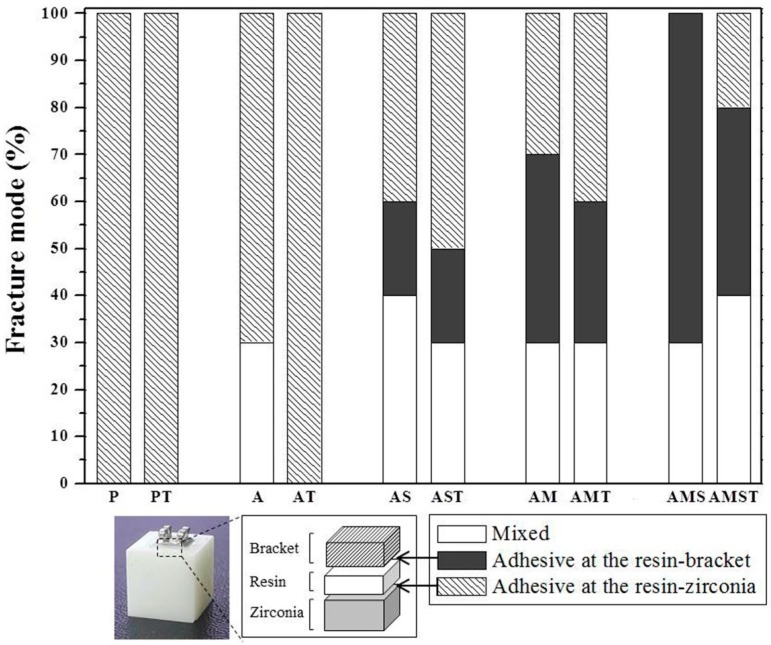
Percentages (%) of the different failure modes after shear bond strength test of an orthodontic metal bracket bonded to Y-TZP.

**Figure 3:**
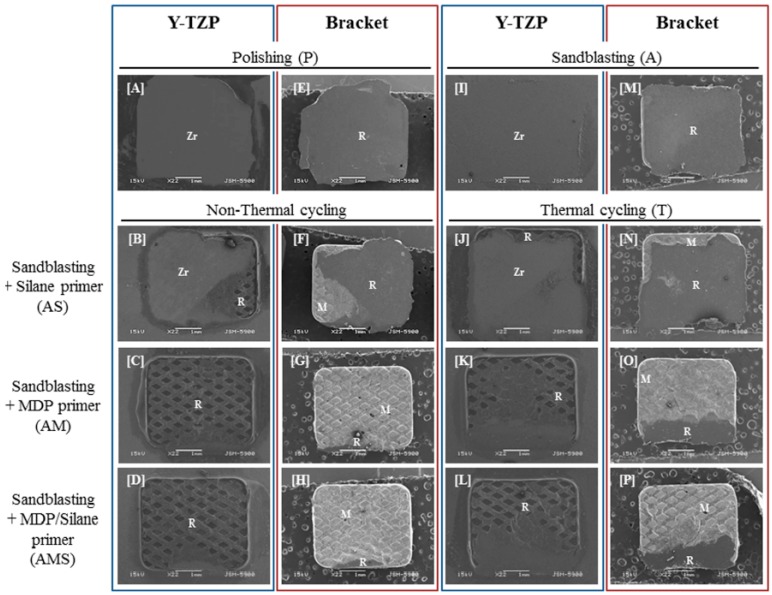
SEM images (22× magnification) of the de-bonded Y-TZP surface (**A**–**D**,**I**–**L**), and the orthodontic metal bracket surface (**E**–**H**,**M**–**P**) after shear bond strength test. Abbreviations: Zr (Y-TZP ceramic), R (Resin cement), M (Orthodontic metal bracket).

The percentages of the different failure modes observed in the bonding area between Y-TZP and the bracket are shown in Figure 2 and SEM images observed in the fractured area are displayed in Figure 3. We confirmed that for the P and PT groups, the resin entirely remained on the bracket (Figure 3E) with a 100% adhesive failure at the zirconia-resin interface. The A group showed 70% adhesive failure at the zirconia-resin interface and 30% mixed failure. The AT group showed 100% adhesive failure similar to the P and PT groups. The AS group showed 20% adhesive failure at the resin-bracket interface, 40% adhesive failure at the zirconia-resin interface, and 40% mixed failure. Compared to the AS group, the adhesive failure of the AST group increased by 50%, and mixed failure decreased by 30%. The AM group showed 40% adhesive failure at the resin-bracket interface, 30% adhesive failure at the zirconia-resin interface, and 30% mixed failure. Compared to the AM group, the adhesive failure of the AMT group increased by 40% at the zirconia-resin interface. The AMS group showed 70% adhesive failure and 30% mixed failure at the resin-bracket interface and the resin entirely remained on the zirconia (Figure 3D). Compared to the AMS group, the adhesive failure of the AMST group decreased by 40%, and 20% adhesive failure occurred at the zirconia-resin interface for this group.

### 3.2. Surface Roughness and Morphology

**Figure 4:**
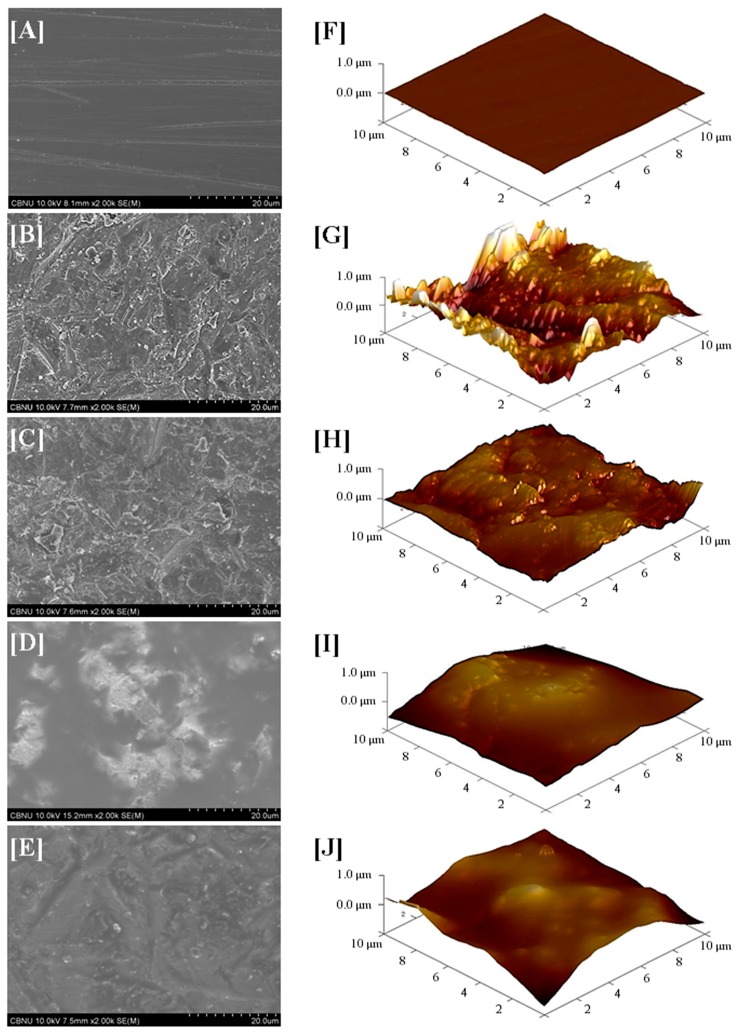
FE-SEM images (2000× magnification) and AFM images (spot size 10 μm × 10 μm). (**A**,**F**) P group; (**B**,**G**) A group; (**C**,**H**) AS group; (**D**,**I**) AM group; (**E**,**J**) AMS group.

The surface roughness values of the surface treated zirconia are shown in Table 3, and the surface morphology is illustrated in Figure 4. The surface roughness values of A, AS, AM, and AMS, except P group, significantly increased (*p* < 0.05). However, there were no significant differences among the surface treated groups (*p* > 0.05). The grooves and bridges of the A, AS, AM, and AMS groups appeared to be distributed irregularly (Figure 4B–E). In particular, the A group with its highest surface roughness value showed the roughest surface (Figure 4G). A very thin layer of primer was observed on the surface of the AS group (Figure 4H), and the surface layer became flatter for the AM and AMS groups as the primer infiltrated into the grooves and bridges formed by alumina sandblasting (Figure 4D,E,I,J).

### 3.3. Surface Characterization

**Figure 5:**
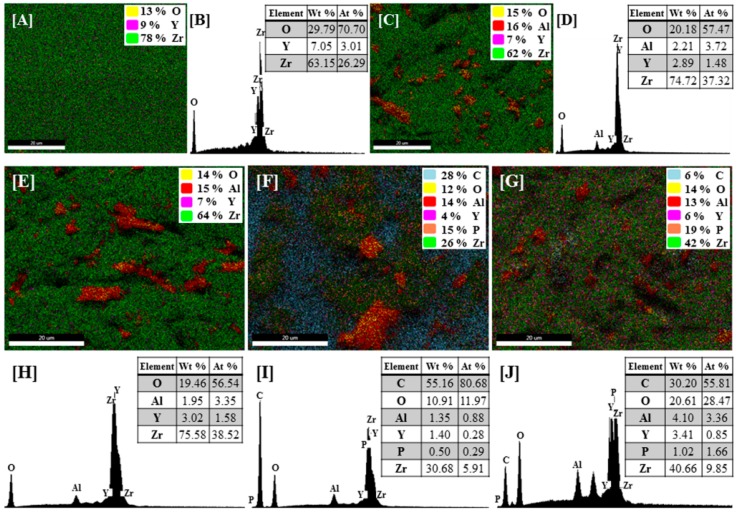
Distribution of elemental composition and EDS detection on the Y-TZP surface of (**A**,**B**) P group; (**C**,**D**) A group; (**E**,**H**) AS group; (**F**,**I**) AM group; (**G**,**J**) AMS group.

The distribution of elemental composition and EDS detection for each group indicated that Al particles remained on the surface after alumina sandblasting for the A, AS, AM, and AMS groups (Figure 5C,E,F,G). C and P were detected on the surface treated with MDP primer and MDP containing silane primer (AM and AMS groups) (Figure 5F,I,G,J).

### 3.4. Chemical Bond Characterization

**Figure 6:**
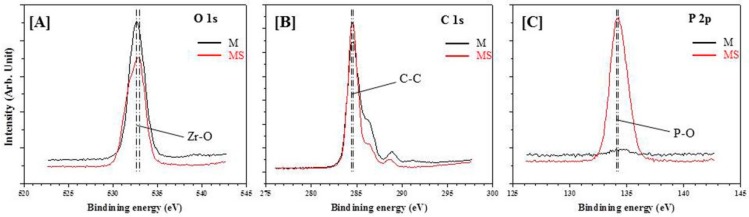
XPS spectra of (**A**) O 1s; (**B**) C 1s; and (**C**) P 2p of the interface between Y-TZP and MDP containing primer (M: MDP primer, MS: MDP containing silane primer).

The chemical bond between the Y-TZP and MDP-based primers (MDP and MDP containing silane primer) was analyzed by XPS (Figure 6). In the O 1s signal, the peaks at 532.58 eV (MDP primer), 532.78 eV (MDP containing silane primer) are contributed to Zr–O–P interactions. These data shows that the between ZrO_2_ and MDP was well formed with a high ratio of Zr–O (Figure 6A). The peaks in the C 1s signal in the MDP primer (284.68 eV) and the MDP containing silane primer (284.58 eV) represent C–C bonds (Figure 6B) and the peaks in the P 2p signal of the MDP primer (134.78 eV) and the MDP containing silane primer (134.28 eV) are assigned to P–O bonds (Figure 6C). The C–C and P–O bonds originate from the internal bonds of MDP-based primers; both these peaks had high intensities in the MDP containing silane primer [41].

## 4. Discussion

In this study, the effect of sandblasting with 110 μm Al_2_O_3_ and the addition of silane, MDP, and MDP containing silane primers on the resin bonding between an orthodontic metal bracket and a Y-TZP ceramic was evaluated. The bond strength was evaluated after surface treatments followed by thermal cycling, which is the most widely used method to evaluate bond durability with the resin. Although there are many different opinions on the number of cycles that need to be used in thermal cycling, it has been mentioned that it is not necessary to increase the number of cycles. This is because, the effect of temperature changes on the bond strength is observed already in the early stage of aging [42]. Other studies have reported that the bond strength decreased with increase in cycle number [14,43]. Based on these reports, this study performed the aging process by thermal cycling up to 5000 times (6 months in vivo) [44] to investigate bond durability with the resin.

Adhesion of an orthodontic bracket to restoratives has a relatively low bond strength, which increases the possibility of a premature failure of the bracket. To solve this problem, the restorative is covered with an orthodontic metal band before it is attached to the bracket. However, band is unaesthetic in the anterior, which requires esthetic compared with the posterior, and may irritate the gingiva. Thus, there is a great interest in developing methods for maintaining bonded brackets directly on restoratives, and appropriate surface treatment methods have been devised [17,18,19].

Some of the evidence shows that Al_2_O_3_ sandblasting, which was performed mainly to compare the different pretreatment conditions of this experiment, is likely to produce defects on the ceramic surface, which depends on the pressure as well as the size of the particles, and decreases the mechanical strength [45,46]. However, it is mentioned that sandblasting not only improves the bond strength by increasing surface roughness, but also removes contaminants on the surface to help the successful bonding of the restoratives [47]. Contaminants in silicon and saliva are typical contaminants in the oral environment. Yang et al. [48] measured the tensile bond strength, and from XPS analysis, proved that sandblasting is effective in removing silicone residues and salivary contaminants. According to Pascal Magne et al. [49], when sandblasting is used with 4-META or MDP-based primers, the bond strength with the resin increases. This is because acidic monomers in the primer react with the oxide groups of zirconia as the surface energy and roughness increase, which improves the wettability. As the bonding between the resin and zirconia is strengthened, it prevents micro-leakage and simplifies complex processes, resulting in reduced treatment time and cost [50]. However, the chemical bond formed by using a primer without sandblasting does not guarantee successful bonding between zirconia and the resin [51].

**Figure 7:**
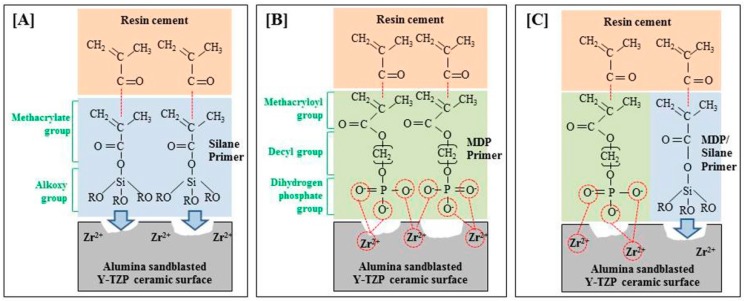
Schematic illustration of micromechanical retention and chemical bonds of the two interfaces, (i) between alumina sandblasted Y-TZP surface and the three different primers; (ii) between the three different primers and Bis-GMA matrix in resin cement; (**A**) Silane primer; (**B**) MDP primer; (**C**) MDP containing silane primer. Sky blue arrows: micro-mechanical interlocking, red line: chemical bonding.

With regard to the bond strength required for adequate bonding of the orthodontic metal bracket, Maijer et al. [52] suggested that the minimum bond strength required for dental clinical use is 5.9–7.9 MPa and Schmage et al. [40] proposed that 6–10 MPa strength is necessary to support normal orthodontic forces. Based on these studies, groups treated with MDP (AM group, 11.92 ± 1.51 MPa) and MDP containing silane primers (AMS group, 13.36 ± 2.31 MPa) after sandblasting were shown to have sufficient bond strength (Figure 1). Groups treated with MDP-based primers had increased roughness after sandblasting as a pretreatment, which enhanced the mechanical strength. XPS analysis showed that the Zr–O bonds contribute to forming chemical bonds between ZrO_2_ (Y-TZP) and MDP (MDP-based primers) (Figure 6A). Additionally, it has been reported that the methacryloyl and the dihydrogen phosphate groups in the MDP-based primer react with the Bis-GMA matrix of the resin and the metal oxides (zirconium oxides, aluminum oxides), respectively (Figure 7B,C), and these reactions improved the chemical bond [53,54].

Some studies showed that sandblasting enhanced the micromechanical interlocking and wettability, which in turn increased the bond strength with the resin [55]. However, the SBS after sandblasting was determined to be 4.98 ± 1.28 MPa in this study, which was higher than the SBS value after polishing; these values are insufficient to support orthodontic forces. The SBS value for the silane primer treatment after sandblasting was 5.13 ± 0.85 MPa, which is not significantly different from the result after only sandblasting. Thus, this treatment did not improve the orthodontic forces (Figure 1) and unlike MDP-based primers, the silane primer does not form a chemical bond with zirconia, but only depends on the mechanical strength due to the micro-unevenness generated during sandblasting (Figure 7A). Silane coupling agents are most commonly used to improve the bond strength between dissimilar materials, namely inorganic (for example, ceramics) and organic (for example, resins). Silane contains methacrylate groups that can react with the resin monomer (polymerization reaction) and the alkoxy group combines with silica-based materials such as porcelain, thereby improving the bond strength (Figure 7A). Therefore, silane based coupling agents cannot be used for non-silica-based restorative materials such as zirconia-based ceramics, alumina ceramics, and metals [56].

This experiment determines the durability of the bond strength with respect to temperature changes in the oral environment by thermal cycling for 5000 cycles at temperatures in the range 5–55 °C. In all the artificially aged groups, the bond strength decreased to such an extent that it could not support orthodontic forces. As suggested by Attia [57], because of the difference in the thermal expansion coefficients of zirconia and the resin during thermal cycling, hoop stress occurs, accelerating the hydrolytic degradation of the resin, thus reducing the bond strength. Moreover, da Silva et al. [37] reported that the bond strength between ceramics treated with MDP-based primers and the resin cement was not maintained when immersed in distilled water for more than six months. However, it has been reported that applying an MDP-based primer to zirconia after thermal cycling did achieve stable bond strength [58]. Similar results were reported by Iwasaki et al. [32], where the bond strength between zirconia treated with MDP primer following 110 μm Al_2_O_3_ sandblasting and the resin cement was measured to be 9.5 MPa, which decreased to 9.2 MPa after thermal cycling, but there was no significant difference. These results are in accordance with the fact that the acidic functional monomer in MDP shows a relatively stable hydrolysis as it contains a long carbonyl chain [5,59]. Based on these reports, thermal cycling for 5000 cycles was performed in this study, which reduced the bond strength of Y-TZP treated with MDP-based primers (MDP, MDP containing silane primer). Thus, it was demonstrated that the hydrolytic degradation of the resin reduced the bond strength.

Failure modes on the bonding surfaces between zirconia and the bracket (Figure 2) reflected the results of the SBS (Figure 1). The groups of polishing, sandblasting, and sandblasting with the silane primer were observed adhesive failure at the zirconia-resin cement interfaces before and after thermal cycling. The failure mode results supported the conclusion that the bond strength between zirconia and the resin was low. The groups that had MDP and MDP containing silane primer coating after sandblasting showed adhesive failures at the resin-bracket interface, which proved the superior bond strength between zirconia and the resin. However, after thermal cycling, the bond strength was reduced by the increased adhesive failure at the zirconia-resin interface.

To improve the bond strength of the Y-TZP based restoratives and the orthodontic metal bracket, Y-TZP was treated with the MDP-based primers after 110 μm Al_2_O_3_ sandblasting, which enhanced resin bond strength. However, all the surface-treated Y-TZP showed a decrease in resin bond strength after 5000 thermal cycles. Thus, further research should focus on highly durable surface treatment methods that help to maintain strong bond strength even after artificial aging.

## 5. Conclusions

In this research, we examined the application of silane, MDP, and MDP containing silane primers, to 110 μm Al_2_O_3_ sandblasted Y-TZP ceramic to study the effects of these primers on the shear bond strength with an orthodontic metal bracket. The following conclusions were drawn:

1. The groups that were treated with the MDP and MDP containing silane primer after alumina sandblasting showed significantly higher SBS values (*p* < 0.05).
2. None of the primers applied to the sandblasted Y-TZP showed durable resin bond strength with the bracket after thermal cycling.

## Figures and Tables

**Table 1:** Groups of the different treated Y-TZP surface in the present study.

Groups	Surface Treatment & Condition (37 °C)	Groups	Surface Treatment & Condition (Thermal Cycling: 5–55 °C)
P	Polishing	PT	Polishing
A	110 μm A1_2_O_3_ Blasting	AT	110 μm A1_2_O_3_ Blasting
AS	110 μm A1_2_O_3_ Blasting + Silane Primer	AST	110 μm A1_2_O_3_ Blasting + Silane Primer
AM	110 μm A1_2_O_3_ Blasting + MDP Primer	AMT	110 μm A1_2_O_3_ Blasting + MDP Primer
AMS	110 μm A1_2_O_3_ Blasting + MDP Containing Silane Primer	AMST	110 μm A1_2_O_3_ Blasting + MDP Containing Silane Primer

**Table 2:** Materials used in the present study.

Material/Trade Name	Main Component	Manufacturer
Y-TZP Ceramic/Zirtooth	88%–96% ZrO_2_, 4%–6% Y_2_O_3_	HASS, Gangneung, Korea
Bracket/Archist Bracket (0.022 twin, Central)	Nickel, Chromium	Daeseung medical, Seoul, Korea
110 μm A1_2_O_3_ Blasting/Rocatec Pre	Aluminium oxide, Size: 110 μm	3M ESPE, St. Paul, MN, USA
Silane Primer/ESPE Sil	3-TMSPMA ^a^, Ethanol	3M ESPE, St. Paul, MN, USA
MDP Primer/Z-PRIME Plus	MDP ^b^, Ethanol	Bisco, Schaumburg, IL, USA
MDP Containing Silane Primer/Clearfil Ceramic Primer	MDP ^b^, 3-TMSPMA ^a^, Ethanol	Kuraray Medical, Tokyo, Japan
Resin Cement/Transbond XT	Silane treated quartz, Bis-GMA ^c^, Bisphenol a bis(2-hydroxyethyl ether) dimethacrylate, Silane treated silica, Diphenyliodonium hexafluorophosphate	3M Unitek, Monorvia, CA, USA

Abbreviations: ^a^ 3-TMSPMA (3-trimethoxysilylpropyl methacrylate), ^b^ MDP (10-methacryloyloxydecyl dihydrogen phosphate), ^c^ Bis-GMA (bisphenol-A-diglycidyl methacrylate).

**Table 3:** Mean surface roughness values (R_a_) after surface treatments.

Groups	Surface Roughness Values (Unit μm)
P	0.037 ± 0.011 ^a^
A	0.546 ± 0.031 ^b^
AS	0.530 ± 0.010 ^b^
AM	0.525 ± 0.041 ^b^
AMS	0.513 ± 0.052 ^b^

^a,b^ Groups shown with different letters were significantly different (*p* < 0.05).
